# Yeast: Translation Regulation and Localized Translation

**DOI:** 10.3390/microorganisms11030739

**Published:** 2023-03-13

**Authors:** Ayala Shiber, Yoav S. Arava

**Affiliations:** Faculty of Biology, Technion-Israel Institute of Technology, Haifa 3200003, Israel

Translation regulation and localized translation are essential for protein synthesis, controlling all aspects of cellular function in health and disease. Studies investigating yeast have provided crucial insights into these processes due to the simplicity of this eukaryotic model organism and its amenability to manipulation. In recent years, it has become a key system for global studies due to the ease of high-throughput screening and availability of large collection libraries of various gene deletions, knockdown, overexpression and fluorophores-fusions. Importantly, processes associated with translation regulation and localized translation are highly conserved in all eukaryotes, from the simple *S. cerevisiae* to highly complex metazoans. 

The collection of articles in this Special Issue of *Microorganisms* presents the power of yeast as a biochemical, genetic and molecular tool to uncover fundamental aspects of Translation regulation and localized translation. Michael Clarke-Whittet and colleagues [1] have used genomic data from different steps of gene expression (from transcription to protein degradation) to derive prediction models of translational feedback loops of autoregulatory RNA-binding proteins. These models allow for a better prediction of protein expression and cooperativity. Neelam Dabas Sen and colleagues [2] have tested the role of the conserved essential DEAD-box RNA helicase Ded1 in translation regulation. The high accessibility of yeast for transcriptomic and ribosome profiling assays allowed them to identify a specific subset of Ded1-dependent mRNAs that responds to stress conditions at the translational level. Moreover, analysis of data derived from deletions strains suggested hyper-dependence on initiation factor eIF4B and, to a lesser extent, eIF4A. Taken together, these results suggest a possible mechanism for Ded1 during stress in translation regulation. A third research article conducted by Ruiz et al. [3] addresses the impact of unregulated amino acids uptake on cellular growth. Here, the utility of yeast for growth assays, together with advanced biochemical assays, uncovered an unknown impact of several amino acids on cellular growth and the possible transporters involved in this sensitivity. The collection also includes two reviews that describe recent data on two important aspects of translation regulation. The first, by Turner and Beilharz [4], deals with mechanisms of alternative polyadenylation, which generate alternative RNA isoforms and consequently affect mRNA translation and localization. Important findings on this process were uncovered in yeast due to the ease of its genetic manipulations and molecular assays. The second review, by Romero et al. [5], focuses on molecular mechanisms of translation regulation by Iron. Intriguingly, while Iron-response mechanisms were among the first translation regulation switches to be discovered in mammals, little is known about the contribution of Iron itself to the different stages of eukaryotic translation. This review therefore provides an overview of the current knowledge in the field and an excellent steppingstone to future studies.

Together, the papers in this Special Issue encompass diverse aspects of translation regulation and localized translation in yeast, from molecular mechanisms to the global view. Thus, the works presented here provide insights for future developments in the field of aspects that are related to yeast physiology, as well as of pathways that are conserved in other organisms.
